# Improved inter-lab precision after CML international scale adoption: a 15-year history of *BCR::ABL1* proficiency testing

**DOI:** 10.1038/s41375-026-03062-6

**Published:** 2026-07-27

**Authors:** Annette S. Kim, Thomas Long, Derek A. Oldridge, Lawrence J. Jennings, Yi Ding, Patricia Vasalos, Christopher Watt, Richard D. Press

**Affiliations:** 1 Department of Pathology, University of Michigan, Ann Arbor, MI USA; 2 Biostatistics Department, College of American Pathologists, Northfield, IL USA; 3 Pathology and Laboratory Medicine, Children’s Hospital of Philadelphia, Philadelphia, PA USA; 4 Department of Pathology, Northwestern University Feinberg School of Medicine, Chicago, IL USA; 5 Department of Pathology, Geisinger Health, Danville, PA USA; 6 Proficiency Testing, College of American Pathologists, Northfield, IL USA; 7 Department of Pathology and Laboratory Medicine, Hospital of the University of Pennsylvania, Philadelphia, PA USA; 8 Department of Pathology and Knight Cancer Institute, Oregon Health and Science University, Portland, OR USA

**Keywords:** Myeloproliferative disease, Health services

## Abstract

Precise inter-laboratory quantitation of *BCR::ABL1* RNA is critical for effective chronic myeloid leukemia (CML) disease management. Although standardized reporting on the International Scale (IS) was specifically developed to improve inter-laboratory reproducibility in *BCR::ABL1* quantitation, the clinical impact of this reporting system has not been comprehensively assessed. We analyzed 15 years of *BCR::ABL1* results (2009–2023) reported by over 200 clinical laboratories (6 annual samples; >5500 results) in a College of American Pathologists proficiency testing program. Inter-laboratory precision was evaluated by standard deviations (SD) for high, intermediate, and low-transcript samples. Early rapid IS adoption over 7 years (8% of labs in 2009; 86% by 2015) was correlated with a progressive decrease in inter-lab SDs of reported *BCR::ABL1* results (high-sample *r* = −0.73, *p* = 0.004; intermediate-sample *r* = −0.71, *p* = 0.022; low-sample *r* = −0.84, *p* = 0.002). IS implementation exceeded 99% by 2019, as inter-lab SD’s reached their nadir value (~0.2). No other assessed variable correlated with time-dependent improvement in inter-lab reproducibility, including reference gene selection (*ABL1* versus others), RQ-PCR reagent selection, assay type (FDA-approved, commercial, or laboratory-developed), or practice setting. IS-based reporting is thus likely the principal driver of enhanced inter-laboratory precision in *BCR::ABL1* quantitation, validating the real-world clinical relevance of adopting robust standardization in clinical molecular diagnostics.

**Figure:**
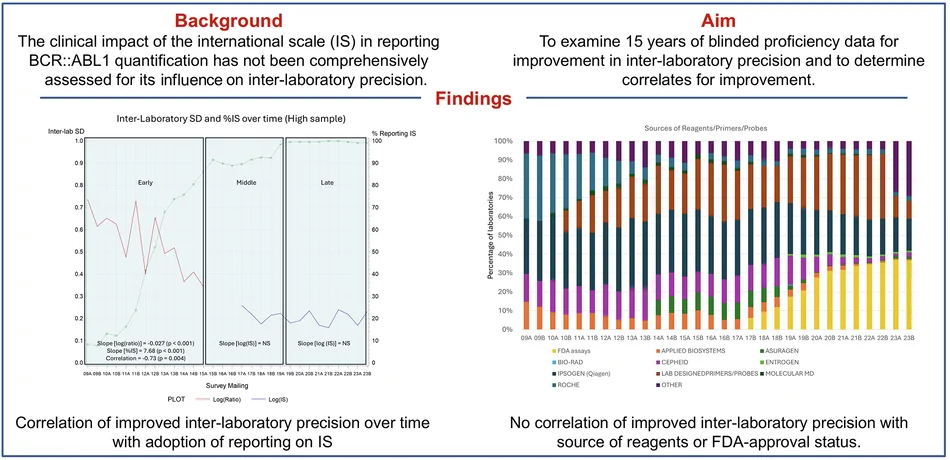

## Introduction

In chronic myeloid leukemia (CML), the *BCR::ABL1* fusion kinase, produced by the Philadelphia chromosome translocation [t(9;22)], serves as both an ideal therapeutic target and a specific diagnostic biomarker of post-treatment measurable residual disease (MRD) [1]. Since the first approval in 2003 of a tyrosine kinase inhibitor (TKI) targeting the *BCR::ABL1* oncoprotein by the Food and Drug Administration (FDA), the long-term survival and prognosis of this once-fatal leukemia have dramatically improved [2]. The extraordinary efficacy of these TKIs demands the use of sensitive, accurate, and precise laboratory methods to quantitatively monitor low-level MRD. Optimal TKI-based management of CML patients with the p210 fusion (created by the e13a2 or e14a2 exon breakpoints) relies on comparison to consensus milestone *BCR::ABL1* RNA levels. These MRD determinations can dictate the choice and/or dose of the specific TKI drug and can serve as a key prognostic marker for predicting long-term outcomes, treatment resistance, and impending relapse [3, 4]. Reverse transcription quantitative PCR (RQ-PCR) remains the primary clinical laboratory method for MRD monitoring, with an analytic measurement range spanning four or more logs. Given the importance of MRD monitoring, inter-laboratory reproducibility in the quantitation of *BCR::ABL1* RNA directly impacts patient management and clinical outcomes [5]. The non-centralized health care system in the United States (US), whereby diagnostic laboratory testing is often performed in different laboratories, depending on the patient’s employment and insurance status, further amplifies this problem.

Despite its well-documented clinical utility, *BCR::ABL1* RQ-PCR remains a technically challenging multi-step laboratory procedure that prompted international efforts early in the TKI treatment era to create a standardized reporting unit for *BCR::ABL1* RNA [6]. In 2008, these efforts resulted in the clinical and analytical validation of the international scale (IS) for the reporting of *BCR::ABL1* RNA levels of the p210 fusion product [7]. IS-based reporting requires the calibration of analytically-determined transcript levels to reference standards tied to clinically proven treatment response thresholds from TKI clinical trials [7, 8].

While the reporting paradigm is standardized through IS alignment, other steps in the *BCR::ABL1* procedure remain non-standardized between laboratories, including RNA extraction, instrumentation, IS calibration methodology, automation *vs* manual methods, and clinical interpretation [9]. Multi-laboratory proficiency testing (PT) allows laboratories to compare the results of their multi-step testing process to gold standard accuracy standards based on the IS and, for some PT vendors, to other participants in aggregate. This PT data also informs the laboratory and clinical community of the inter-laboratory reproducibility. According to the Clinical Laboratories Improvement Act (CLIA), some form of PT is required of US-based clinical diagnostic laboratories. The College of American Pathologists (CAP) is the leading provider of standardized PT materials and has surveyed laboratories on *BCR::ABL1* transcript quantitation since 2006. The resulting CAP-sponsored PT data is the most comprehensive, unbiased, real-world source of laboratory performance data for different analytes, including many that are based on quantitative PCR [10–17].

The purpose of this study was to evaluate whether inter-laboratory precision of quantitative *BCR::ABL1* RNA reporting has improved over time, and, if so, whether that improvement is attributable to the institutional adoption of IS-based standardized calibration. This study reviews 15 years of serial data from the CAP proficiency testing survey, whereby over 200 clinical laboratories blindly tested three samples with varying *BCR::ABL1* (p210) transcript levels twice per year (annual A and B mailings for this survey; 2009–2023, 30 total mailings, 90 total samples). This dataset should be representative of the performance status of *BCR::ABL1* (p210) testing across the primarily US-based clinical laboratory community during the timeframe when labs were progressively implementing IS-based reporting, allowing for a comprehensive assessment of the role of IS in inter-laboratory reproducibility.

## Methods

### Survey information

Fifteen years of CAP proficiency testing data on quantitative *BCR::ABL1* (p210) testing were examined, spanning the 2009A through 2023B mailings. Although originally an educational PT program, the *BCR::ABL1* MRD PT survey became a graded survey in 2014, tying laboratory performance with laboratory accreditation. Laboratory performance was scored based on reported %IS and MR (log molecular response) responses, where all reported data had to fall within +/– 2 standard deviations from the mean MR value of all participants. By this definition, ~5% of laboratory responses would “fail” in each mailing. Laboratories reporting persistent outlier results on this CAP survey risk formal lab licensure penalties under CLIA regulations, including cessation of clinical testing. Any *BCR::ABL1* (p210) result that was not aligned to the IS and/or calculated using a non-*ABL1* control gene could not be graded.

The individual mailings of the *BCR::ABL1* MRD PT survey included additional questions about the laboratory practices of the participants. For instance, each survey mailing queried laboratories on the selection of a control gene. In addition, many survey mailings interrogated the origin of the PCR primers and, by the 2017B mailing, were specifically asking if the laboratory’s assay was (or was not) FDA approved (modified or unmodified), with the option to specify the assay vendor.

### Specimen information

Blinded proficiency specimens were provided to participants as RNA extracted from a CML cell line diluted in *BCR::ABL1* negative RNA to achieve the final dilution. Each participant received three samples per mailing, one mimicking a new CML diagnosis sample (“high” level of *BCR::ABL1* transcript), and two additional samples at “intermediate” and “low” transcript levels. For the analyses described herein, these different dilutions were binned into categories of “high” (10-fold dilution), “intermediate” (1: 100, 1:1000, and 1:5000) or “low” (1:10,000, 1:50,000, and 1: 100,000) *BCR::ABL1* RNA for each mailing. Some mailings included negative control specimens not used in the analysis, and those mailings only had two (not three) levels of specimens. Specimen dilutions for each mailing are presented in Supplementary Table 1.

### Response information

Participant responses were expressed as relative transcript levels on a logarithmic scale [log(ratio)] from 2009A through 2015A (“ratio” defined as p210 RNA divided by reference gene RNA), and as log (IS) (*BCR::ABL1* international scale) from 2012A through 2023B. Responses were also recorded as relative log reduction from the “diagnostic sample” (highest *BCR::ABL1* sample of the blinded 3-sample mailing) from 2009A through 2018A, and as molecular response [MR, equal to log_10_(100/%IS)] from 2018B through 2023B. For several years, there was an overlap of reporting mechanisms, and participants could report results in a variety of formats. Where this was true, all relevant responses are included in this study. During 2009-2013, the transcript level of the “high” sample was not directly interrogated as the relative log-reduction level was zero, leaving no “high” data for those mailings. IS and MR-based reporting started in 2012A and 2018B, respectively, but only became mandatory in 2018B. Before the mandatory reporting of %IS and MR, there were periods (2011B–2018B) where a participating laboratory could report both log(ratio) and %IS, or log reduction and MR.

### Data analysis

Response data were excluded for log ratio values of 0, IS values of 0, or any response with a qualifier (e.g., non-numeric value above or below a laboratory’s detection limit, “not detected,” or “negative” IS value). MR responses that were greater than 0.4 from an MR value calculated by CAP based upon other response data (*n *= 70) were removed as presumed typographical or calculation errors rather than erroneous results. A 2- pass 3-SD (standard deviation) outlier screen was applied for each measure, level, and mailing before data were summarized and analyzed.

For each response measure, transcript level, and mailing, the inter-laboratory SD was calculated and plotted against mailing date to visualize the time trend. A secondary overlaid axis displayed the percentage of participants reporting IS-aligned data. For the three time windows [early (2009-2015A), middle (2015B-2019A), and late (2019B-2023B)], a time-dependent regression line of the SD was fit, and a Pearson’s Correlation Coefficient (R) was calculated between SD and percentage of participants reporting IS at each level. Statistical tests were performed for time-dependent slopes of the fitted SD regression lines, slopes of the % reported IS regression lines, and the significance of the correlations for each time window and level. ANOVA was used to compare means between laboratories using *ABL1* and any other non-*ABL1* control gene. Levene’s Homogeneity of Variance test compared inter-laboratory variances (1) between *ABL1* and non-*ABL1* control gene utilization, (2) between FDA and non-FDA assay utilization, (3) between commercial (non-FDA) and laboratory-developed assays, and (4) between academic and reference laboratories. All analyses were completed using SAS version 9.4 (Cary, NC).

### Ethics approval and consent to participate

All methods were performed in accordance with relevant guidelines and regulations. This study analyzed de-identified, aggregate laboratory performance data from College of American Pathologists proficiency testing/survey programs using contrived proficiency testing materials. The authors did not receive patient-level data, identifiable private information, identifiable biospecimens, or laboratory-identifiable performance data. Therefore, the study does not qualify as human subjects research as there are no human participants, interaction or intervention with individuals, or identifiable human material or data. Accordingly, ethics committee approval and informed consent to participate were not applicable and were not obtained.

## Results

### Participant and survey information

Although CAP began providing the *BCR::ABL1* (p210) MRD PT survey in 2006, the data from the first few years of this survey were returned to CAP in a myriad of different formats and units. This early data also preceded the 2008 publication defining the standardized IS for reporting quantitative *BCR::ABL1* (p210) levels [7]. This study, therefore, evaluates the CAP PT survey data from the 2009A to the 2023B mailings, during which participant laboratories grew from 48 to 228. This study encompasses 30 mailings (two mailings, A and B, per year) with a total of 75 samples and 5,534 laboratory PT responses (Supplementary Table 1).

### Percent of laboratories using IS over time

**Fig. 1:**
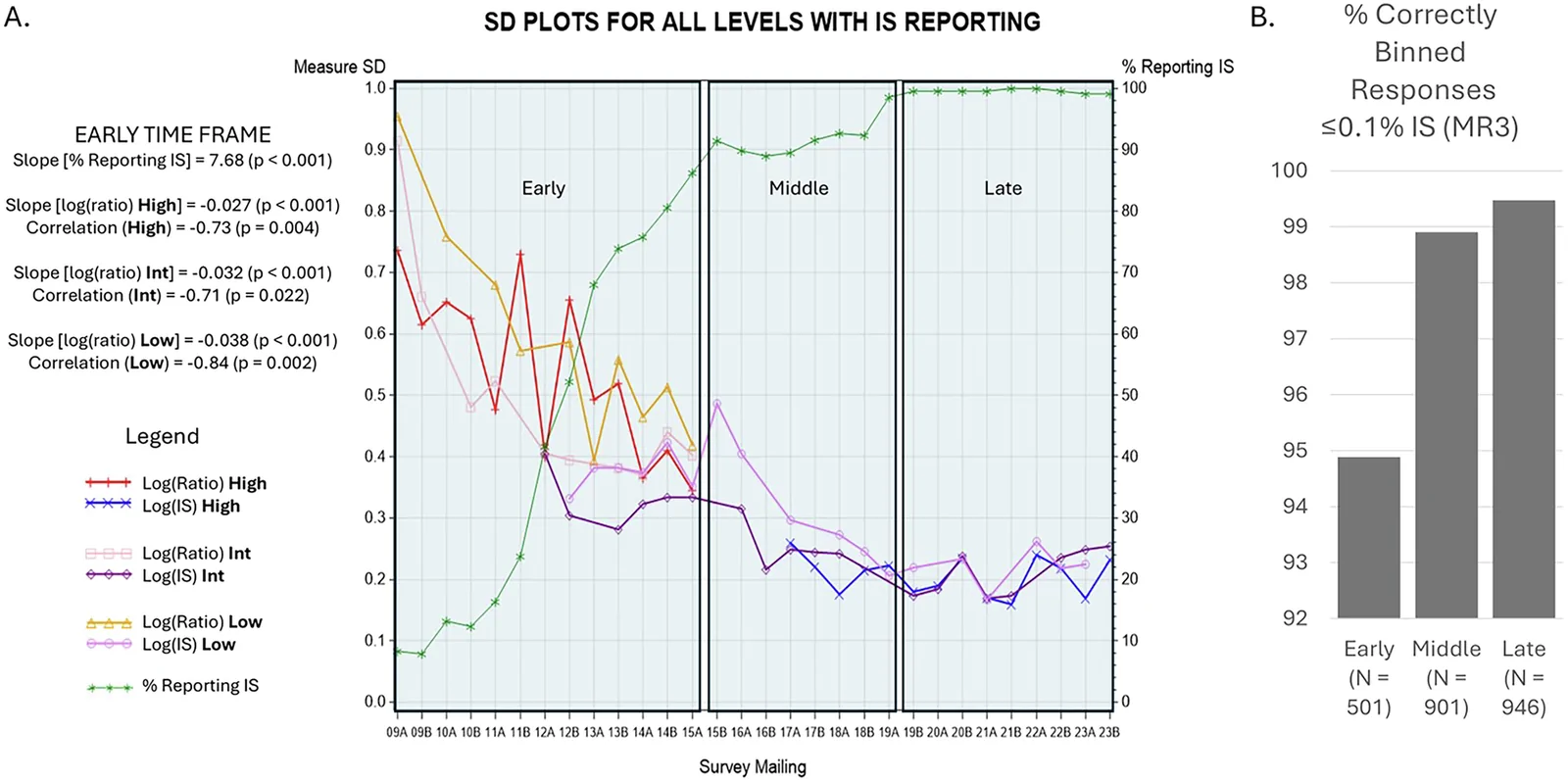
Performance of laboratories in *BCR::ABL1* quantification over time. **A** Inter-laboratory standard deviation of *BCR::ABL1* transcript quantification reported as log(ratio) and Log(IS) over time compared to percent of laboratories reporting on IS. The graph presents data for the high transcript sample (red/plus and blue/X solid lines), the intermediate transcript sample (pink/square and purple/diamond lines), and the low transcript sample (yellow/triangle and lavender/circle lines). The percentage of labs reporting on the International Scale is overlaid (green/asterisk line). Time windows are indicated by the light blue boxes: [early = 2009A – 2015A; middle = 2015B – 2019A; late = 2019B – 2023B]. The slope and correlation coefficients are depicted for the early time windows. Abbreviations: Int intermediate, IS International Scale, SD standard deviation. **B** Percentage of respondents that correctly identified samples whose transcript levels were ≤0.1% IS, representing a major molecular response, separated by time periods of the study. MR molecular response.

After the 2008 publication describing standardized *BCR::ABL1* reporting on the IS, CAP began requesting IS-based PT survey results reporting for laboratories using this standardized reporting unit [7]. Utilization of IS-based reporting by CAP-participating labs (which requires calibration and quantitative quality control) increased from 8% in 2009 to 86% in 2015 (Fig. 1; Supplementary Fig. 1), at which point CAP mandated IS-based reporting for formal PT survey grading. By 2019B, over 99% of laboratories reported in IS. To determine if this time-dependent increase in IS-based reporting correlated with other *BCR::ABL1* performance metrics, the 15 years of PT data were divided into “early” (2009-2015A), “middle” (2015B-2019A), and “late” (2019B–2023B) time windows.

### Inter-laboratory reproducibility (Precision) over time correlates with the adoption of IS

A major force driving standardization of *BCR::ABL1* reporting on IS was the heterogeneity of testing methods between labs, limiting inter-laboratory comparability of results. To directly assess whether the primary goal of the IS standardization effort—improved inter-laboratory precision— was realized, the inter-laboratory SD of blinded CAP PT survey results for each of the 30 mailings was examined. Since the precision of quantitative testing of analytes correlates with their abundance and low-level targets generally have higher imprecision, 3 distinct “levels” of *BCR::ABL1* RNA per CAP mailing were defined: “high”, “intermediate”, and “low” (Supplementary Table 1) [18]. Due to the non-IS results reported in the early years of this study, the inter-laboratory SDs were calculated using two log-transformed reporting units: *BCR::ABL1*/control gene RNA ratio [log(ratio)] (used in 2009–2015) and/or %IS (used after 2012 for the low and intermediate samples, and after 2017 for the high-level sample). Inter-laboratory coefficients of variation (CV, SD/mean) were also calculated for the lowest transcript samples (Supplementary Fig. 2).

For all three *BCR::ABL1* RNA levels (high, intermediate, and low), the inter-laboratory precision improved over time during the 2009–2015A “early ” time frame, demonstrated by a significant time-dependent decrease in the inter-lab SD of the reported log(ratio) *BCR::ABL1* result (slope = −0.027, −0.032, −0.038 for high, intermediate, and low *BCR::ABL1* samples, respectively; *p* < 0.001 for all three; Fig. 1A, left panel; Table 1). The percentage of labs reporting on the IS rose dramatically during this “early” period (slope = 7.68, *p* < 0.001, Fig. 1A, left panel; Table 1). In this early time frame, the time-dependent increase in labs using IS was significantly inversely correlated with the decrease in the inter-laboratory log-ratio SD (high sample *r* = −0.73, *p* = 0.004; intermediate sample *r* = −0.71, *p* = 0.022; low sample *r* = −0.84, *p* = 0.002; Fig. 1A; Table 1).

**Table 1:** Slopes of log(ratio) SD, log(IS) SD, and % labs on IS along with their correlation.

Time-Window	*BCR::ABL1* transcript level (all participants)	Log(ratio) or Log(IS) SD slope	*p* value of slope	Slope of the percent of labs on IS	Correlation with the slope of the percentage of labs on IS	*p* value of correlation
Early (2009A–2015A)	High	−0.027	**<0.001**	7.68 (*p* < 0.001)	−0.73	**0.004**
	Intermediate	−0.032	**<0.001**		−0.71	**0.022**
	Low	−0.038	**<0.001**		−0.84	**0.002**
Middle (2015B–2019A)	High	NS	0.31	0.89 (*p* = 0.003)	NS	0.68
	Intermediate	NS	0.2		NS	0.94
	Low	−0.037	**<0.001**		NS	0.23
Late (2019B–2023B)	High	NS	0.41	NS (*p* = 0.30)	NS	0.97
	Intermediate	0.009	**0.003**		NS	0.06
	Low	NS	0.61		NS	0.39

Time- Window	(IS only participants)					
(2012–2019A)	High	NA	**NA**	3.19 (*p* < 0.001)	NA	NA
	Intermediate	−0.01	**<0.001**		−0.69	**0.018**
	Low	−0.012	**0.003**		NS	0.37
Late (2019B–2023B)	High	NS	0.41	NS (*p* = 0.30)	NS	0.97
	Intermediate	0.009	**0.003**		NS	0.06
	Low	NS	0.61		NS	0.39

The upper table includes all lab participants, while the lower table includes only labs reporting on the IS (2012 and beyond).

*NS* not significant.

NA data not available (no IS data available for the “High” transcript level from 2012–2016).

By 2015, at the start of the “middle” time frame (2015B-2019A), IS-based reporting had already approached its near-100% maximum (with 91.5% of laboratories reporting on IS by 2015B), and thus increased less steeply (slope = 0.89; *p* = 0.003) than in the previous early time frame (Fig. 1A; Table 1). The concomitant inter-laboratory SD for *BCR::ABL1* log(IS) mirrored this pattern in this middle period, with a minimal decline over time toward its ultimate late period 0.2 log nadir (Fig. 1A; Table 1). Specifically, the high and intermediate level *BCR::ABL1* samples showed no statistically significant decrease in log(IS) SD over this “middle” time period (slope not different from zero), and there was then no statistically significant correlation between inter-lab SD (of log(IS) values) and IS-based reporting (Fig. 1A; Table 1). Only the low-level *BCR::ABL1* sample continued to show a significantly declining inter-lab SD during this middle time period [middle time slope = −0.037 (*P* < 0.001), Table 1], although the SD decline was not significantly correlated with the concomitant slight increase in IS-based-reporting (Table 1).

By the start of the “late” time window (2019B-2023B), the inter-laboratory precision had already reached its nadir value of ~0.2 log(IS) SD for all three *BCR::ABL1* RNA levels, and the log(IS) SDs did not further decrease during this time frame (slopes no longer significantly negative) (Fig. 1A, right panel, Table 1). Similarly, by 2019, utilization of IS-based reporting had already plateaued at >99%, with no further increase (slope not significantly different than zero). There was no significant statistical correlation between inter-laboratory reproducibility and IS-based reporting in this “late” time period (Table 1).

Since the “early” and “middle” time periods included a mix of laboratories reporting and not reporting IS-based results, a separate analysis of only laboratories reporting on the IS between 2012 and 2019A was performed. During this period, the inter-laboratory SDs for log(IS) values trended significantly downward for the intermediate (slope = −0.010, *p* < 0.001) and low (slope = −0.012, *p* = 0.003) *BCR::ABL1* samples (Table 1). Concomitantly, IS-based reporting significantly increased during this 2012–2019 time period (slope =3.19; *p* < 0.001). [This evaluation was not performed on the high transcript sample since CAP did not collect IS results for the high transcript samples until 2017, when approximately 90% of laboratories were reporting on the IS.] In comparison, after 2019, there was no analogous downward trend in log(IS) SD values (slope not significantly less than zero), nor any significant correlation with IS-based reporting (which had plateaued).

Clinically, the correct assessment of key milestone reduction of transcript levels is essential for appropriate patient care. The clinical milestone of a major molecular response (MMR, defined as ≤0.1% IS, or MR3) was evaluated to assess what percentage of respondents correctly binned the proficiency samples as having achieved an MMR for samples with “low” transcript levels. The percentage of respondents correctly identifying MMR samples demonstrating ≤0.1% IS progressively rose, from 94.9% of participants correctly identifying an MMR in the early timeframe, to 98.9% in the middle timeframe, and to 99.5% in the late timeframe (Fig. 1B). Other molecular milestones (>10% IS, 1–10% IS, and 0.1–1% IS could not be evaluated due to the values of the proficiency samples falling too close to the milestone thresholds and technical variability (as much as 5-fold for inter-laboratory reproducibility [7] and 3-fold for intra-laboratory reproducibility [5]) limiting that assessment.

### Control gene over time

**Fig. 2:**
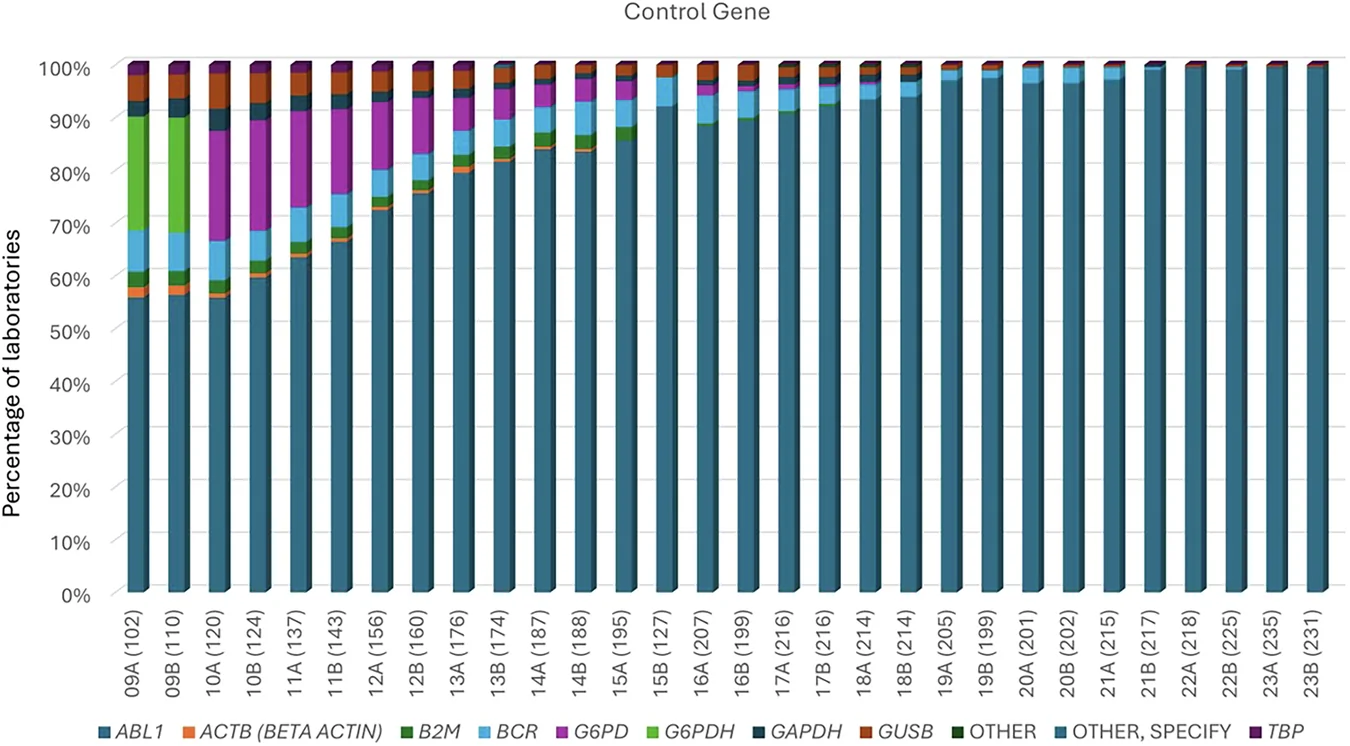
Percentage of laboratories using various control genes over time. Parenthetic values depict the number of participating laboratories.

Since the first publication describing optimized control genes for *BCR::ABL1* (p210) RQ-PCR testing, *ABL1* has been identified as a control gene that does not significantly differ in expression between normal and leukemic samples at diagnosis [19]. *ABL1* is one of only three control genes suitable for the IS conversion/calibration and is the most commonly used reference gene in clinical labs [6]. During this 15-year study, the utilization of *ABL1* as a control gene increased from 55.9% of laboratories (2009A) to 99.6% by 2023B (Fig. 2).

### Inter-laboratory reproducibility (precision) over time does not correlate with control gene utilization

To assess if the time-dependent increase in *ABL1* utilization as a control gene correlated with improved inter-laboratory reproducibility (precision), a homogeneity of variance comparison was performed between laboratories using *ABL1* as the control gene and laboratories using any non-*ABL1* control gene. To minimize the effects of IS-based calibration on this analysis, only *BCR::ABL1* MRD mailings prior to 2012B, when CAP began collecting IS-based reporting data, were included. These early mailings offer statistically robust comparisons, since the number of laboratories using a non-*ABL1* control gene became very small thereafter. In this “early” period (2009A to 2012A), characterized by a steep time-dependent reduction in inter-laboratory SDs of *BCR::ABL1* log-ratios (slopes = −0.034, −0.076, −0.072 for the high, intermediate, and low samples, respectively), there were no mailings or samples for which the inter-lab BCR::ABL1 precision significantly differed between *ABL1* and non-*ABL1* laboratories.

### Sources of primers/probes over time

**Fig. 3:**
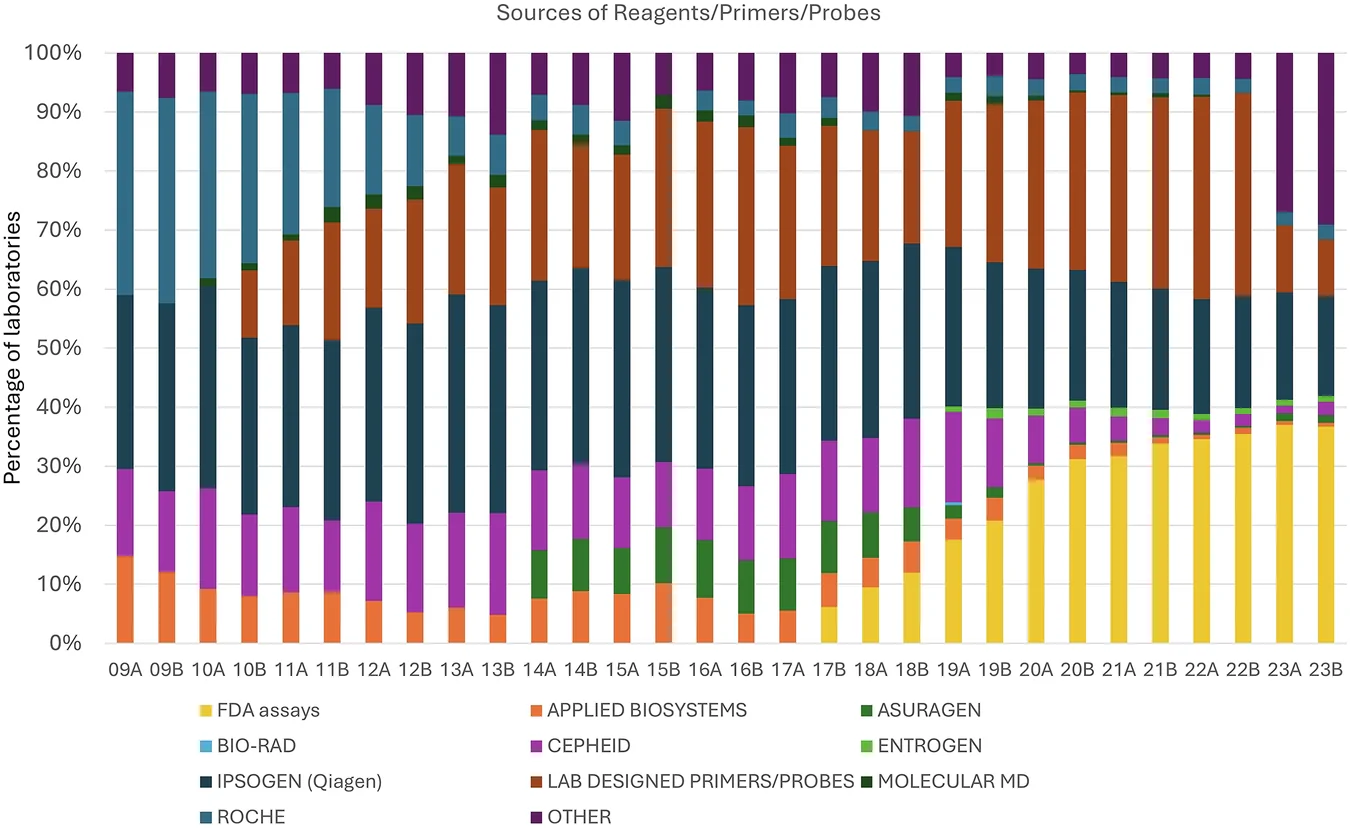
Reported source of assay reagents, primers, and/or probes over time. Different commercial assay vendors are represented by different colors with their distribution per survey mailing. FDA-approved assays are in yellow, starting in use during the 17B survey mailing.

**Fig. 4:**
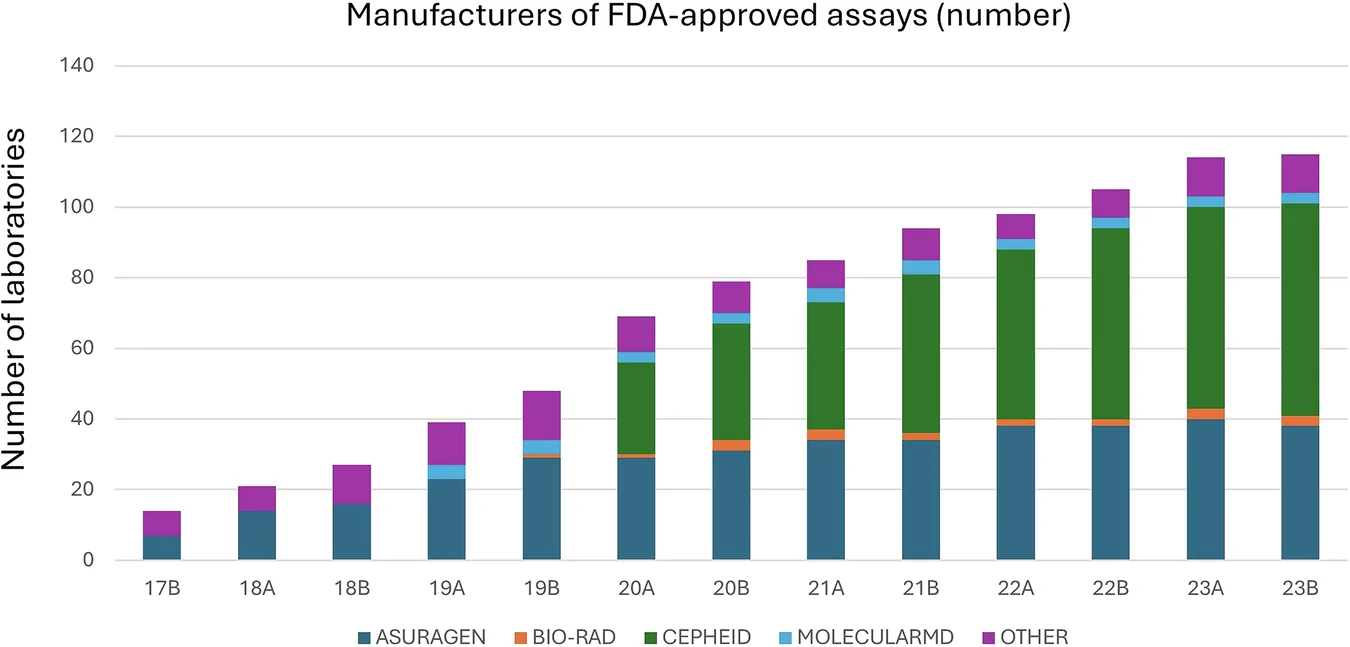
Manufacturers of FDA-approved assays over time. The specific providers (vendors) of the FDA-approved assays from Figure 3 are delineated here over time per survey mailing.

Participating laboratories have historically chosen from a wide range of sources for the primers and probes used in laboratory-developed *BCR::ABL1* (p210) RQ-PCR tests, without dominance of a single manufacturer or reagent source (Fig. 3). There was no apparent change in sourcing of reagents throughout the 2009–2023 monitoring period. Commercially packaged assays that changed ownership during this time period were grouped together. Commercial assays that were approved as an in vitro diagnostic by the Food and Drug Administration (FDA) during the study period were grouped as FDA assays once they gained FDA approval (Fig. 3, yellow: see also Fig. 4).

The first FDA-cleared quantitative *BCR::ABL1* (p210) test became available in 2016. Since 2017, CAP PT mailings specifically asked laboratories if FDA-approved methods for *BCR::ABL1* quantification were used. As of the data freeze for this manuscript, three additional *BCR::ABL1* assays have also attained FDA authorization. From 2017 to 2023, laboratories’ use of FDA-approved (unmodified or modified) *BCR::ABL1* assays rose from 6% to over 30% of participants (Figs. 3 and  4).

### Inter-laboratory reproducibility (precision) over time does not correlate with the implementation of an FDA-approved assay or commercial (versus non-commercial) sources of primers and probes

Inter-laboratory precision for *BCR::ABL1* RNA levels had already reached its nadir (~0.2 log SD) by the time FDA-approved assays (gold bars, Fig. 3) became available starting in 2016, suggesting no causal relationship between these two variables. Nonetheless, a homogeneity of variance comparison between inter-laboratory precision (SD) and the use of an FDA-approved assay was performed. Across all survey mailings and transcript levels (high, intermediate, and low), there was no statistically significant difference in the inter-lab *BCR::ABL1* precision between FDA and non-FDA laboratories. In addition, among laboratory-developed tests, the comparison of laboratories using laboratory-developed primers and probes (brown stacked bars, Fig. 3) compared to a commercial kit (non-brown, excluding FDA-approved kits, Fig. 3) was also examined. The use of commercial, as opposed to non-commercial primers and probes, similarly did not correlate with improved reproducibility across all survey mailings and transcript levels.

### Inter-laboratory reproducibility (precision) over time does not correlate with academic versus reference designation of laboratories

As part of the ordering information for all PT kits, laboratories must specify if they identify as hospital/medical center, hospital/medical center- academic, independent/commercial reference lab, non-hospital site, or physician office (Supplementary Table 2). Laboratories that self-identified as either academic or reference laboratories represented the two largest groups. Analyzing only the declared academic versus reference laboratory designations, across all survey mailings and transcript levels (high, intermediate, and low), there was no statistically significant difference in the inter-lab *BCR::ABL1* precision between academic and reference laboratories. In addition, across all time points and all levels, there was no statistically significant difference in the *BCR::ABL1* transcript level result means between the academic and the combined non-academic laboratories.

## Discussion

The IS was developed with the specific goal of improving inter-laboratory reproducibility for *BCR::ABL1* p210 RQ-PCR quantitation. To address whether this goal was achieved, this study evaluated over 5534 blinded PT results from over 200 laboratories over 15 years, encompassing 30 individual semi-annual mailings and 75 blinded RNA samples. This cohort of laboratories, chosen only by their decision to participate in CAP PT in compliance with mandatory PT requirements, thus represents the broad heterogeneity of clinical molecular diagnostic laboratories in the US and is likely less enriched for laboratories with CML-specific research interests, who may have been over-represented in other published *BCR::ABL1* inter-laboratory sample-sharing studies [18, 20, 21]. To our knowledge, this study utilizes the largest inter-laboratory sample-sharing data set to date for *BCR::ABL1* RQ-PCR testing. The major conclusion of this study is unequivocal: inter-laboratory precision has significantly improved over time with increased adoption of IS-based reporting. In particular, during the “early” years (2009–2015), with the largest year-over-year increase in IS adoption, there was a statistically significant inverse correlation between increased IS-based reporting and decreased inter-laboratory SDs of reported RQ-PCR testing results across all levels of *BCR::ABL1* p210 transcript analyzed, confirming a robust time-dependent association between these 2 variables.

From 2009–2015, laboratories reporting on the IS increased from 8% to 86%, while the inter-laboratory SD decreased from approximately 0.7–0.9 to 0.2 log(IS), indicating approximately 7 years to high-level adoption of IS from the time of the original IS publication in 2008 [7]. After 2019, however, when IS-based reporting approached 100%, there was no further improvement in inter-laboratory SD (for any *BCR::ABL1* sample level), suggesting that IS-based reporting could be causally linked to improved inter-laboratory precision. In this “late” time window, inter-laboratory SD values stabilized at 0.2 log(IS), a level similar to findings from other studies restricted to laboratories using the IS [22]. When only IS-reporting laboratories before 2019 were analyzed in our data set, there was only a subtle time-dependent improvement in inter-laboratory precision. This declining SD slope for IS-only laboratories is not as steep as when non-IS results were included, as expected, potentially reflecting the gradual adjustment of laboratories to the appropriate application of IS standardization.

Although these data definitively show a significant correlation between IS-based reporting and improved inter-laboratory precision, causality cannot be definitively proven. Other possible explanations for the observed time-dependent decrease in inter-lab SD were considered. The increased time-dependent use of the *ABL1* gene as the reference gene for RQ-PCR, despite its many drawbacks as a control gene, also increased over time from 56% of labs in 2009, to 75% in 2012, and to 99% in 2023 [9]. However, there was no evidence of any significant effect of the use of the *ABL1* reference gene (versus any non-*ABL1* reference gene) on the inter-lab reproducibility of *BCR::ABL1* measurements for the “early” years (2009A-2012A) when there were sufficient non-*ABL1* laboratories to assess. This analysis suggests that the increased time-dependent use of the *ABL1* reference gene is not a major contributor to the observed improvement in inter-laboratory precision.

The convergence upon a single technical method for performing *BCR::ABL1* RQ-PCR is also unlikely to account for the observed improvement in inter-laboratory precision, given the wide range of different methods/kits/reagents used for RQ-PCR throughout the study period, without any time-dependent dominance of a single method. An additional possible explanation for the improved inter-lab SDs could be the increased utilization of FDA-approved assays compared to laboratory-developed assays. While the adoption of FDA-approved *BCR::ABL1* assays did rise during the 2017–2023 time period, the inter-lab standard deviations of RQ-PCR testing results had already reached their nadir value by 2017. In addition, there was no significant difference in inter-laboratory SDs between users of laboratory-developed tests (LDTs) and FDA-approved assays. This data demonstrates that FDA approval does not indicate improved assay performance, a conclusion also supported by other comparative assay performance studies [11–13]. Similarly, there was no overall significant difference in the inter-laboratory reproducibility of laboratories using a commercial (non-FDA-approved) kit versus laboratory-designed primers and/or probes. In addition, there was no statistical difference in the overall inter-laboratory precision when comparing academic and reference laboratories.

In short, the time-dependent improvement in inter-laboratory precision observed in this study does not correlate independently with variables such as reference gene choice, PCR primer/probe source, use of FDA-approved kits, use of commercial kits, or performance of the testing in either an academic or reference laboratory. Instead, the improved precision strongly correlates with the increased use of IS-based reporting. This finding is clinically impactful given the consensus treatment implications for CML patients achieving critical *BCR::ABL1* transcript level milestones during TKI treatment, such as early molecular response (*BCR::ABL1* < 10% IS), major molecular response (MMR) (*BCR::ABL1* < 0.1% IS), deep molecular response (*BCR::ABL1* < 0.01%), or molecular relapse (1-log increase in *BCR::ABL1*) [3]. In fact, this improvement in reproducibility over time correlated with the increased percentage of respondents who correctly identified transcript levels meeting criteria for an MMR over time.

This study has several limitations. First, laboratories self-reported their use of the IS, which was not independently verified and may be misclassified. Second, data capture for laboratory methods evolved over the course of the 15-year study period, leading to heterogeneous, inconsistent documentation across the survey years. Third, participation in this CAP PT program was voluntary (although participation in some form of PT is mandatory under CLIA), introducing the potential for selection bias. Fourth, laboratories could enter or exit participation in this PT survey at will, resulting in a dynamic and shifting cohort over time. Fifth, while one might speculate that CAP surveys could preferentially retain higher-performing laboratories, the substantial time-dependent increase in participants suggests that increasing numbers of newer, less experienced laboratories were also included, potentially balancing or diluting this effect. Lastly, missing data points were common due to changing laboratory participation and reporting procedures, which could impact the completeness and interpretation of certain analyses.

In summary, this study provides a comprehensive 15-year analysis of quantitative *BCR::ABL1* (p210) RQ-PCR proficiency testing across a broad spectrum of clinical diagnostic laboratories. This large study of blinded PT results provides strong evidence that (1) inter-laboratory precision of *BCR::ABL1* results has markedly improved in the past 15 years, and (2) this improvement in inter-laboratory precision correlates with, and is likely attributable to, the progressive adoption of the IS for results reporting. This study also demonstrates that neither standardization of control gene usage (particularly adoption of *ABL1*) nor the eventual introduction of FDA-approved assays had a significant impact on inter-laboratory precision, compared to the effect of IS implementation. By 2019, with nearly universal use of the IS by over 200 participating laboratories, inter-laboratory variance reached a nadir of 0.2 log(IS) SD. These findings underscore the critical role of IS-based reporting in ensuring consistent and reliable molecular monitoring in CML, enabling better patient care through more reproducible laboratory measurements.

## Supplementary information

Supplementary Materials

## Data Availability

The data analyzed for this study are the property of the College of American Pathologists (CAP), and specific laboratory submission data is proprietary to ensure privacy. Summaries of the de-identified data are presented in this manuscript. Requests for de-identified data should be addressed to the CAP.
