# Supplementary material for: Improved inter-lab precision after CML international scale adoption: a 15-year history of BCR::ABL1 proficiency testing
Source: Leukemia. 2026 Jul 27;40(10):2236–43. doi: 10.1038/s41375-026-03062-6 (PMC13612228; doi:10.1038/s41375-026-03062-6)
Supplement: Supplementary file 1 [file 41375_2026_3062_MOESM1_ESM.docx]

**Supplemental Table 1.** Participant and survey information

| Survey | Total number of participants | Number reporting on %IS (%) | Transcript type | "high" dilution | "intermediate" dilution | "low" dilution | Negative Sample |
| --- | --- | --- | --- | --- | --- | --- | --- |
| 2009A | 48 | 8.3 | e14a2 | neat | 1:100 | 1:10,000 |  |
| 2009B | 51 | 7.8 | e14a2 | neat | 1:1,000 |  | Negative |
| 2010A | 53 | 13.2 | e14a2 | neat |  | 1:10,000 | Negative |
| 2010B | 57 | 12.3 | e14a2 | neat | 1:100 |  |  |
| 2011A | 61 | 16.4 | e13a2 | neat | 1:100 | 1:10,000 |  |
| 2011B | 63 | 23.8 | e14a2 | neat |  | 1:10,000 | Negative |
| 2012A | 77 | 41.6 | e13a2 | neat | 1:1,000 |  |  |
| 2012B | 90 | 52.2 | e14a2 | neat | 1:1,000 | 1 :100,000 |  |
| 2013A | 103 | 68.0 | e14a2 | neat |  | 1:10,000 | Negative |
| 2013B | 115 | 73.9 | e14a2 | neat | 1:100 | 1:100,000 |  |
| 2014A | 132 | 75.8 | e14a2 | 1:10 | 1:100 | 1:10,000 |  |
| 2014B | 133 | 80.5 | e13a2 | 1:10 | 1:1,000 | 1:100,000 |  |
| 2015A | 145 | 86.2 | e13a2 | 1:10 | 1:1,000 | 1:10,000 |  |
| 2015B | 106 | 91.5 | e13a2 | 1:10 |  | 1:100,000 | Negative |
| 2016A | 176 | 89.8 | e13a2 | 1:10 | 1:1,000 | 1:100,000 |  |
| 2016B | 171 | 88.9 | e14a2 | 1:10 | 1:100 |  | Negative |
| 2017A | 181 | 89.5 | e13a2 | 1:10 | 1:100 | 1:10,000 |  |
| 2017B | 190 | 91.6 | e14a2 | 1:10 | 1:1,000 |  | Negative |
| 2018A | 190 | 92.6 | e14a2 | 1:10 | 1:1,000 | 1:100,000 |  |
| 2018B | 195 | 92.3 | e13a2 | 1:10 |  | 1:10,000 | Negative |
| 2019A | 199 | 98.5 | e13a2 | 1:10 |  | 1:10,000 | Negative |
| 2019B | 193 | 99.5 | e14a2 | 1:10 | 1:100 | 1:100,000 |  |
| 2020A | 197 | 99.5 | e14a2 | 1:10 | 1:1,000 |  | Negative |
| 2020B | 197 | 99.5 | e13a2 | 1:10 | 1:100 | 1:10,000 |  |
| 2021A | 214 | 99.5 | e14a2 | 1:10 | 1:100 | 1:10,000 |  |
| 2021B | 215 | 100.0 | e14a2 | 1:10 | 1:5,000 |  | Negative |
| 2022A | 214 | 100.0 | e13a2 | 1:10 |  | 1:50,000 | Negative |
| 2022B | 222 | 99.5 | e13a2 | 1:10 | 1:100 | 1:5,000 |  |
| 2023A | 233 | 99.1 | e14a2 | 1:10 | 1:5,000 | 1:10,000 |  |
| 2023B | 228 | 99.1 | e13a2 | 1:10 | 1:5,000 |  | Negative |

Definitions: neat – neat concentration of RNA from the CML cell line. All dilutions in this table are represented as fold-dilutions from the neat RNA concentration. For mailings (2010B and 2012A) where both dilutions would be considered “Intermediate,” only one level was included in the analyses (other not shown in this table). The exception is the 2022B mailing where the 1:5,000 dilution was binned with the “low” levels. The samples that were negative for transcript are listed in the table but were not included in the analyses in the manuscript.

**Supplemental Table 2.** Participant self-reporting of laboratory type/practice setting.

| **LABTYPE** | **Frequency** | **Percent** | **Cumulative Frequency** | **Cumulative Percent** |
| --- | --- | --- | --- | --- |
| **Hospital/Medical Center** | 1141 | 26.50 | 1141 | 26.50 |
| **Hospital/Medical Center Lab - Academic (Univ or Teaching)** | 1497 | 34.77 | 2638 | 61.26 |
| **Independent/Commercial Reference Lab** | 1243 | 28.87 | 3881 | 90.13 |
| **Non Hospital Site** | 411 | 9.54 | 4292 | 99.67 |
| **Physician Office Lab/Clinic** | 14 | 0.33 | 4306 | 100.00 |
| **Frequency Missing = 1228** | | | | |

**Supplemental Figure 1**. Percentage of laboratories reporting on the International Scale over time. X axis lists the mailing designations with the number of total participants in parentheses.

**Supplemental Figure 2**. CV plot for the most dilute (low transcript) levels [log (ratio) in red and log(IS) in blue] plotted against the % participants reporting in IS (green/asterisks). Abbreviations: CV – coefficient of variance; IS – international scale, LOGRATIOL_CV – the CV of the most dilute specimen (low transcript level) as measured as log ratio (negative numbers).
